# Genome-wide identification and expression analysis of the cucumber *PYL* gene family

**DOI:** 10.7717/peerj.12786

**Published:** 2022-01-11

**Authors:** Zeyu Zhang, Shilei Luo, Zeci Liu, Zilong Wan, Xueqin Gao, Yali Qiao, Jihua Yu, Guobin Zhang

**Affiliations:** 1 State Key Laboratory of Aridland Crop Science, Gansu Agricultural University, Lanzhou, China; 2 College of Horticulture, Gansu Agricultural University, Lanzhou, China

**Keywords:** Abscisic acid, Cucumber, *PYL* gene, Abiotic stress

## Abstract

Abscisic acid (ABA) is a very important hormone in plants. It regulates growth and development of plants and plays an important role in biotic and abiotic stresses. The Pyrabactin resistance 1-like (PYR/PYL) proteins play a central role in ABA signal transduction pathways. The working system of *PYL* genes in cucumber, an important economical vegetable (*Cucumis sativus* L.), has not been fully studied yet. Through bioinformatics, a total of 14 individual *PYL* genes were identified in Chinese long ‘9930’ cucumber. Fourteen *PYL* genes were distributed on six chromosomes of cucumber, and their encoded proteins predicted to be distributed in cytoplasm and nucleus. Based on the phylogenetic analysis, the *PYL* genes of cucumber, *Arabidopsis*, rice, apple, *Brachypodium distachyon* and soybeancould be classified into three groups. Genetic structures and conserved domains analysis revealed that *CsPYL* genes in the same group have similar exons and conserved domains. By predicting cis-elements in the promoters, we found that all *CsPYL* members contained hormone and stress-related elements. Additionally, the expression patterns of *CsPYL* genes were specific in tissues. Finally, we further examined the expression of 14 *CsPYL* genes under ABA, PEG, salt stress. The qRT-PCR results showed that most *PYL* gene expression levels were up-regulated. Furthermore, with different treatments about 3h, the relative expression of *PYL8* was up-regulated and more than 20 times higher than 0h. It indicated that this gene may play an important role in abiotic stress.

## Introduction

ABA is one of the hormones that regulates growth and development of plants, participating in plant response to biological and abiotic stresses (Lee & Luan, 2012). The study found that abscisic acid not only participates in plant growth, such as seed dormancy, cell division and elongation, stomatal movement, embryo development, but also in response to biotic and abiotic stresses (Bartels & Sunkar, 2005; Finkelstein, Gampala & Rock, 2002). PYLs belong to the START (Star-related lipid-transfer) superfamily of ligand-binding proteins, which contains hydrophobic ligand cavity and can bind directly to ABA (Grill & Himmelbach, 1998; Szostkiewicz et al., 2010). ABA signal transduction pathway mainly consists of three parts: ABA receptor PYR/PYL/PCAR, negative regulator factor protein phosphatase PP2C (Ma et al., 2009; Park et al., 2009) and positive regulator factor protein kinase SnRK2 (Kobayashi et al., 2005). Normally PP2C binds to SnRK2, SnRK2 serine/threonine residues are dephosphorylated, protein kinases are inactivated, and the ABA signaling pathway is silenced (Ma et al., 2009; Vlad et al., 2009). When stimulated by external signals, ABA content increased and closely combined with the internal hydrophobic cavity of PYR / PYL / RCAR protein. This binding is accomplished by the formation of ion pairs between a lysine residue side chain of the receptor protein in the cavity and the carboxyl group of the ABA. After ABA combined with the receptor protein, the conformation of the receptor protein in hydrophobic cavity is changed. It is mainly reflected in two conservative rings, “gate ring” and “latch ring” (Fujii et al., 2009). After ABA binding, a position of the proline residue on the “gate ring” was shifted and to close the cavity, and a serine residue on the ring was squeezed out of the cavity by ABA, then a histidine residue on the “latch ring” is transferred into the cavity and binds to the ABA through van der Waals force and hydrogen bond, finally the “latch ring” conformation changes locked the “gate ring”, leads to ABA fixed in the cavity, and provides a binding site for the PYL protein and the PP2C (Szostkiewicz et al., 2010). The activity of PP2C bound with PYL was inhibited, the SnRK2 is separated from the PP2C, and then the downstream transcription factors are phosphorylated and ABA signaling pathway is initiated.

ABA and its required proteins are found in algae, but they lack ABA receptor and do not respond to ABA. Terrestrial plants are considered to be monophyletic and gradually evolve into ABA activated receptors in continuous evolution (Sun et al., 2019). The PYR/PYL/RCAR family contains 14 genes in *Arabidopsis* (Dupeux et al., 2011). According to phylogenetic tree, ABA receptors have been divided into three subfamilies: I, II, and III (Ma et al., 2009). Subfamilies I and II receptors are monomeric, whereas subfamily III receptors are dimeric and exclusive to the more recently diverged angiosperms (Umezawa et al., 2010). Subfamily III receptors require ABA for dimer dissociation, which results in low basal receptor activity in the absence of ABA (Dupeux et al., 2011). Furthermore, orthologous genes in other crops have been reported, including 13 *PYLs* in rice, 13 *PYLs* in maize, 27 *PYLs* in cotton, 14 *PYLs* in tomato, and 8 *PYLs* in grape (Yadav et al., 2020; He et al., 2018; Chen et al., 2017; González-Guzmán et al., 2014; Boneh et al., 2012). Moreover, the functions of some *PYL* genes have been successfully verified. For example, the *AtPYL6* and *AtPYL13* in *Arabidopsis* have been shown to inhibit seed germination (Fuchs et al., 2014), *AtPYR1*, *AtPYL1*-*5*, *AtPYL8* can promote ABA induce seed germination, stomatal closure and root growth (Gonzalez-Guzman et al., 2012; Zhao et al., 2014). In addition to the *PYL* genes associated with growth, *AtPYL4*, *AtPYL9* can improve the drought resistance of *Arabidopsis* (Pizzio et al., 2013; Zhao et al., 2016). Overexpression of *OsPYL5* and *OsPYL10* in rice can enhance drought resistance, salt tolerance and cold resistance (Kim et al., 2014). Moreover, the ectopic expression of *OsPYL3* in *Arabidopsis* can enhance the cold tolerance and drought tolerance of *Arabidopsis* (Lenka et al., 2018). Overexpression of *ZmPYL8*, *ZmPYL9* and *ZmPYL12* in maize can enhance the cold resistance of maize (He et al., 2018). Excessive expression of *GhPYL9*-*11A* in cotton enhances drought resistance of cotton, and overexpression of *GhPYL9*-*11A* in transgenic *Arabidopsis* is sensitive to ABA during seed germination and early seedling stage (Chengzhen et al., 2017). These results indicated that *PYL* genes play a significant role in plants tolerance under abiotic stress. However, the information of *PYL* gene family in cucumber has not been reported.

Cucumber (*Cucumis sativus* L, 2n = 2X = 14) has a small number of chromosomes and small genome (Luming et al., 2012). The first assembly of the genome sequence has been completed (Guo et al., 2009). Cucumber is an annual vine or climbing herb of Cucurbitaceae Cucumis, which is one of the important vegetable crops in China. After long-term cultivation and domestication, it is widely distributed all over the world. During the process of cucumber cultivation, it is vulnerable to various biotic and abiotic stresses, which affect quality and yield. Therefore, this study identified and analyzed the cucumber *PYL* gene family and used quantitative PCR (qRT- PCR) to determine the expression of 14 cucumber *PYL* genes under ABA, salt stress and drought stress, which provided a theoretical basis for the future study of cucumber stress resistance.

## Materials and Methods

### Plant materials and treatment

The germinated seeds (Cucumis sativus L, “L306” cultivar) were treated by 5% NaClO 10 min and deionized water washing 3–5 times. After that, the cucumber seeds were put into an artificial climate incubator to promote germination. The cultivation condition: relative humidity 80%, temperature 25 °C/18 °C (day/night), and the light intensity during the day is 250 µmol m^−2^ s^−1^. After 4 days of culture, cucumber seedlings were soaked in Yamazaki nutrient solution for hydroponics. Cucumber seedlings were treated when they grew to three true leaves. This experiment set up four treatments (T1: 50 µmol/L ABA, T2: 100 *μ*mol/L ABA, T3: 200 mmol/L NaCl, T4: 10% PEG). The cucumber leaves were collected after 0 h, 3 h, 6 h, 12 h and 24 h. Finally, put fresh samples in liquid nitrogen, immediately and stored in −80 °C refrigerator.

### Genome-wide identification of cucumber *PYL* gene family

The protein sequences of 14 *PYL* genes in *Arabidopsis* were downloaded from the *Arabidopsis* information resource (TAIR) website (https://www.arabidopsis.org/). And then, the BLASTp comparison was carried out in cucumber genome data (http://cucurbitgenomics.org/), the retrieval threshold was set as *E*-value < E^−10^ (Altschul et al., 1997). Scaffold assemblies of three cucumber lines (Chinese long ‘9930’, Gy14, and B10) are available so far. This draft genome was assembled using Chinese long inbred line ‘9930’ version 3 (Cavagnaro et al., 2010). The login number of duplicate genes in the comparison results was manually deleted. Then the gene domain after screening was searched in the Pfam database (http://pfam.xfam.org/search#tabview=tab1). A total of 15 *CsPYL* genes were obtained. The Polyketide_cyc2 domains (PF10604) were found in all *CsPYLs*. After naming according to the chromosome order, it was found that *CsPYL1* had an unknown domain and a large molecular weight, so it was removed. Then downloading HMM files for HMM search comparison, a total of 14 *PYL* genes family members in cucumber were identified. Finally, through NCBI-CDD database (https://www.ncbi.nlm.nih.gov/cdd/) (Marchler-Bauer et al., 2015) identified whether the candidate sequences have PYR/PYL (RCAR) like domain (cd07821).

### Analysis of chromosome location and collinearity analysis

The Chinese long ‘9930’ V3 version of gff3 file was downloaded from cucumber genome database, and the chromosome location and chromosome length information of 14 *PYL* genes were screened out and named *CsPYLs* according to their distribution on chromosomes. The MG2C (http://mg2c.iask.in/mg2c_v2.0/) was used to map the chromosomal location. Using Mcscanx search for homology, the protein-coding genes from cucumber genome was compared against itself and *Arabidopsis* genomes using BLASTp and retrieval threshold was set as *E*-value < E^−5^. Others were not modified by default parameters. Whole-genome BLASTP results were used to compute collinear blocks for all possible pairs of chromosomes and scaffolds (Wang et al. 2012a). Subsequently, TBtools was used to highlight the identified *PYL* collinear pairs and their collinear pairs with *Arabidopsis* (Chen et al., 2020).

### Selective pressure analysis of cucumber *PYL* gene family

PAL2NAL (http://www.bork.embl.de/pal2nal/index.cgi?) were used to calculate the non-synonymous/synonymous (d_N_/d_s_) value of duplicate gene pairs (Goldman & Yang, 1994).

### Sequence analysis and basic information of cucumber *PYL* gene family

The subcellular localization of PYL protein in cucumber was predicted by Wolf PSORT (https://wolfpsort.hgc.jp/) (Xiong et al., 2015). The number of amino acids, isoelectric point (PI), molecular weight (MW) and other physical and chemical information of *CsPYL* genes were identified on the ExPASy website (https://web.expasy.org/protparam/) (Artimo et al., 2012). Additionally, the secondary structure of cucumber PYL protein were predicted through the online web site (https://npsa-prabi.ibcp.fr/cgi-bin/npsa_automat.pl?page=/NPSA/npsa_sopma.html).

### Construction of phylogenetic tree

The phylogenetic tree of *PYL* gene family of cucumber*, A. thaliana,* rice*,* soybean*,* grape*, B. distachyon* and apple were constructed by using MEGA 7 software with muscle methods to align multiple sequences (Kumar, Stecher & Tamura, 2016). The amino acid substitution model was (JTT+G) (Supplemental Information 3), and the bootstrap values were calculated for 1,000 replicates. Finally, a stable minimum neighbor tree was selected to represent their evolutionary relationship and beautify the constructed tree by ITOL website (Ivica & Peer, 2016) (http://itol.embl.de/).

### Analysis of gene exon-intron structures and protein conserved motifs

Upload CDS and genome-wide sequences of 14 cucumber *PYL* genes on GSDS (GSDS v2.0) websites (http://gsds.gao-lab.org/) (Yang et al., 2015) for genetic structure analysis; The conserved motifs of 14 cucumber *PYL* genes were analyzed by MEME website (http://meme-suite.org/tools/meme) (Bailey et al., 2009). The maximum motif number was set to 10, and the remaining parameters were the default values. Functional annotations of these motifs were performed using HHpred (https://toolkit.tuebingen.mpg.de/tools/hhpred) (Zimmermann et al., 2018).

### Analysis of cis-acting elements in *CsPYL* gene promoters

In order to predict the cis-acting elements in the promoter of *CsPYL* genes, TBtools was used to extract the genomic sequences of 14 cucumber *PYL* genes up to 2,000 bp upstream of the initiation codon (ATG). Then, the 2,000 bp sequences of 14 *CsPYLs* were submitted to the online website plantcare for prediction (http://bioinformatics.psb.ugent.be/webtools/plantcare/html/) (Lescot, 2002).

### Transcriptome analysis of *CsPYL* genes in different tissues

In order to study the gene-specific expression of *CsPYL* genes in different tissues of cucumber, the accession number PRJNA80169 was used from cucumber genome data (http://cucurbitgenomics.org/) to obtain cucumber RNA samples from different tissues and organs (tendril-base, tendril, root, leaf, stems, ovary-unfertilized, ovary-fertilized, ovary) RNA-Seq data (Li et al., 2011; Harris et al., 2008). Finally, the method of log2 was used for data conversion and Tbtools software was used to draw the expression heat map of *CsPYL* gene.

### RNA extraction and real-time RT-PCR

Total RNA was isolated from collected samples using a Plant RNA Extraction kit (Tiangen, China). The complementary DNA was synthesized by using fastking cDNA dispersing RT supermaxs kit (Tiangen, China) with 2 µl RNA as template. The CDS sequences of *CsPYL* genes were input into the homepage of Shanghai biology company (Shanghai, China) for online primer design (Table S1), and then the primer sequences were synthesized. The SYBR Green kit (Tiangen, China) was used for fluorescence quantitative analysis. The volume of reaction system was 20 µL, which contained 2 µL cDNA solution, 10 µL2^∗^SuperReal PreMix Plus, 0.6 µL of 10 µM forward and reverse primers, 0.4 µL 50^∗^ROX Reference Dye and 6.4 µL of distilled deionized water. Then the qRT-PCR was performed with LightCycler^^®^^ 480 II real-time fluorescence quantitative PCR instrument. The amplification program conditions were as follows: 95 °C for 15 min, and 40 cycles of 95 °C for 10 s and 60 °C for 30 s. Each sample was replicated 3 times. The relative expressions of *CsPYL* genes were calculated by 2^−ΔΔCT^ method (Livak & Schmittgen, 2002), with CsActin as an internal control (Gan et al., 2013; Wan et al., 2010). Each sample repeat at least three times.

SPSS 20.0 was used for one-way ANOVA, and Duncan method was used to test the significance of the difference (*P* < 0.05) and data were all the average of 3 replicates. The Excel was used to complete the histogram of relative expressions.

## Results

### Genome-wide analysis and chromosome distribution of *CsPYL* gene family

We determined that 14 putative *CsPYL* were present in the cucumber genome through BLASTP by using 14 *AtPYL* sequences as references. From the analysis of their physical and chemical properties (Table 1), *PYL2* has the largest number of amino acids and the largest molecular weight of protein. The other members of *PYL* protein have 162∼233 amino acids and protein molecular weight range 18.17 from 25.66 KDa. In addition, isoelectric points (PI) are from 4.91 to 8.28. Only *CsPYL4* is basic protein, and the others are acidic proteins. The total average hydrophobic index of 14 *CsPYL* genes family members were less than zero, all of them were hydrophilic proteins.

**Table 1:** Genomic information and protein characteristics of 14 *CsPYLs* gene family members in cucumber.

**Gene ID**	**Gene name**	**Chromosome**	**Gene location**		**Amino acid number**	**Molecular** **weight** **(kd)**	**PI**	**Instability index**	**Aliphatic index**	**Grand average of hydropathicity**
			**Start position**	**End position**						
CsaV3_2G030780.1	PYL1	2	20234462	20235706	162	18.17	5.43	43.43	72.22	−0.246
CsaV3_2G030790.1	PYL2	2	20234462	20239446	344	38.47	5.25	37.41	79.01	−0.168
CsaV3_3G001740.1	PYL3	3	1311481	1312909	229	25.70	5.41	49.19	80.87	−0.519
CsaV3_3G008140.1	PYL4	3	7005104	7008146	185	20.81	8.28	39.57	87.84	−0.391
CsaV3_3G022000.1	PYL5	3	19093676	19095745	184	20.58	5.97	47.35	100	−0.217
CsaV3_3G033450.1	PYL6	3	28742374	28744464	206	22.59	6.44	48.23	85.05	−0.274
CsaV3_3G049190.1	PYL7	3	40090988	40092469	232	25.47	5.35	32.61	78.97	−0.306
CsaV3_4G026530.1	PYL8	4	15776156	15778129	233	25.66	7.73	46.6	81.93	−0.359
CsaV3_4G035560.1	PYL9	4	25042643	25043922	193	21.59	5.47	41.14	81.76	−0.473
CsaV3_5G000620.1	PYL10	5	319763	324420	195	21.89	6.44	36.06	94.87	−0.423
CsaV3_5G008890.1	PYL11	5	5494966	5495652	179	19.90	4.91	34.88	84.92	−0.158
CsaV3_5G010560.1	PYL12	5	6531332	6532063	243	25.86	6.79	50.96	82.22	−0.187
CsaV3_6G001990.1	PYL13	6	1361372	1362257	208	23.02	6.59	41.55	90.19	−0.067
CsaV3_7G025250.1	PYL14	7	14609356	14611908	236	26.69	6.66	43.33	94.07	−0.088

**Table 2:** Secondary structure and subcellular localization of 14 cucumber *CsPYLs* gene family members.

**Gene ID**	**Gene name**	**α-helix %**	**Beta turn %**	**Random coil %**	**Extended strand %**	**Subcellular localization**
CsaV3_2G030780.1	PYL1	37.04	6.17	38.27	18.52	Cytoplasm, Nucleus
CsaV3_2G030790.1	PYL2	30.23	8.72	39.53	21.51	Cytoplasm, Nucleus
CsaV3_3G001740.1	PYL3	38.43	5.68	39.74	16.16	Cytoplasm, Nucleus
CsaV3_3G008140.1	PYL4	39.46	5.41	37.30	17.84	Cytoplasm
CsaV3_3G022000.1	PYL5	41.30	5.98	35.87	16.85	Cytoplasm
CsaV3_3G033450.1	PYL6	29.13	6.80	43.20	20.87	Cytoplasm, Chloroplast
CsaV3_3G049190.1	PYL7	40.95	8.19	35.34	15.52	Cytoplasm
CsaV3_4G026530.1	PYL8	35.9	3.85	44.02	16.24	Cytoplasm, Chloroplast
CsaV3_4G035560.1	PYL9	40.41	5.18	35.23	19.17	Nucleus, Cytoplasm
CsaV3_5G000620.1	PYL10	36.41	4.62	41.54	17.44	Nucleus, Cytoplasm
CsaV3_5G008890.1	PYL11	46.37	3.91	31.84	17.88	Nucleus
CsaV3_5G010560.1	PYL12	35.8	7.00	40.33	16.87	Chloroplast
CsaV3_6G001990.1	PYL13	29.81	6.25	39.90	24.04	Cytoplasm, Chloroplast
CsaV3_7G025250.1	PYL14	42.37	3.39	34.75	19.49	Cytoplasm, Chloroplast

Subcellular localization prediction display (Table 2) that most of the *PYL* genes in cucumber were predicted to be located in cytoplasm and nucleus, while *CsPYL6, CsPYL8, CsPYL12, CsPYL13* and *CsPYL14* were located in chloroplast. The results of secondary structure prediction (Table 2) found that the secondary structure of most *CsPYL* proteins were mainly composed of α-helix and irregular curl, and the proportion of extended structure and β-angle were the smallest.

**Figure 1:**
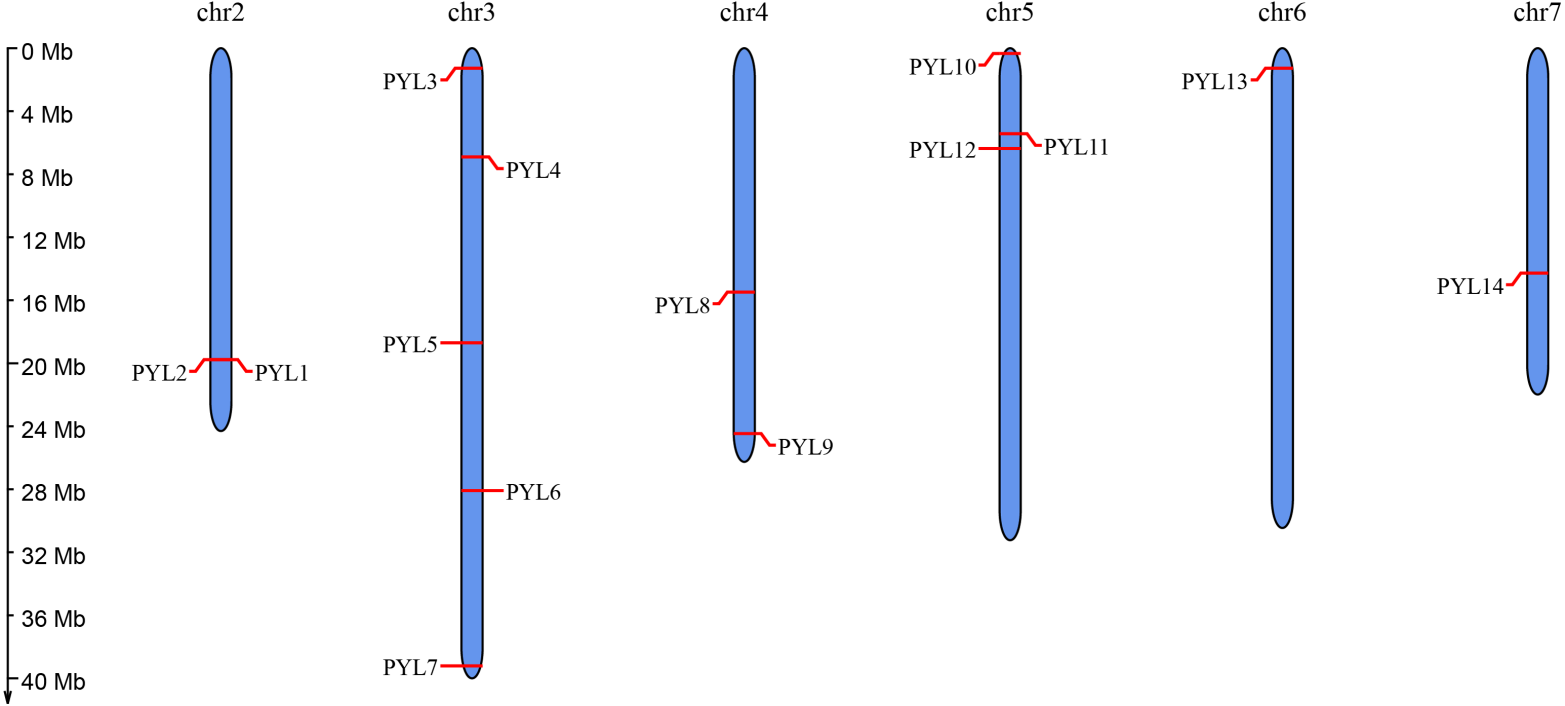
Chromosomal distribution and localization of *CsPYLs*. The chromosome names are shown at the top of each chromosome. The chromosome scale is in millions of bases (Mb) on the left.

Moreover, to predict the position of *CsPYLs* gene on the chromosome information, using the online website MG2C to map the chromosomal location. The distribution of *PYL* genes on chromosomes showed that 14 members of *PYL* gene family were distributed on 6 chromosomes of cucumber (Fig. 1). There were more *PYL* genes on chromosomes 3 (*PYL3*-*7*) and 5 (*PYL10*-*12*). Both *CsPYL1* and *CsPYL2* were located on the same site of Chr 2 and belonged a pair of tandem duplication genes.

### Collinearity analysis of the *PYL* gene family in cucumber

**Figure 2:**
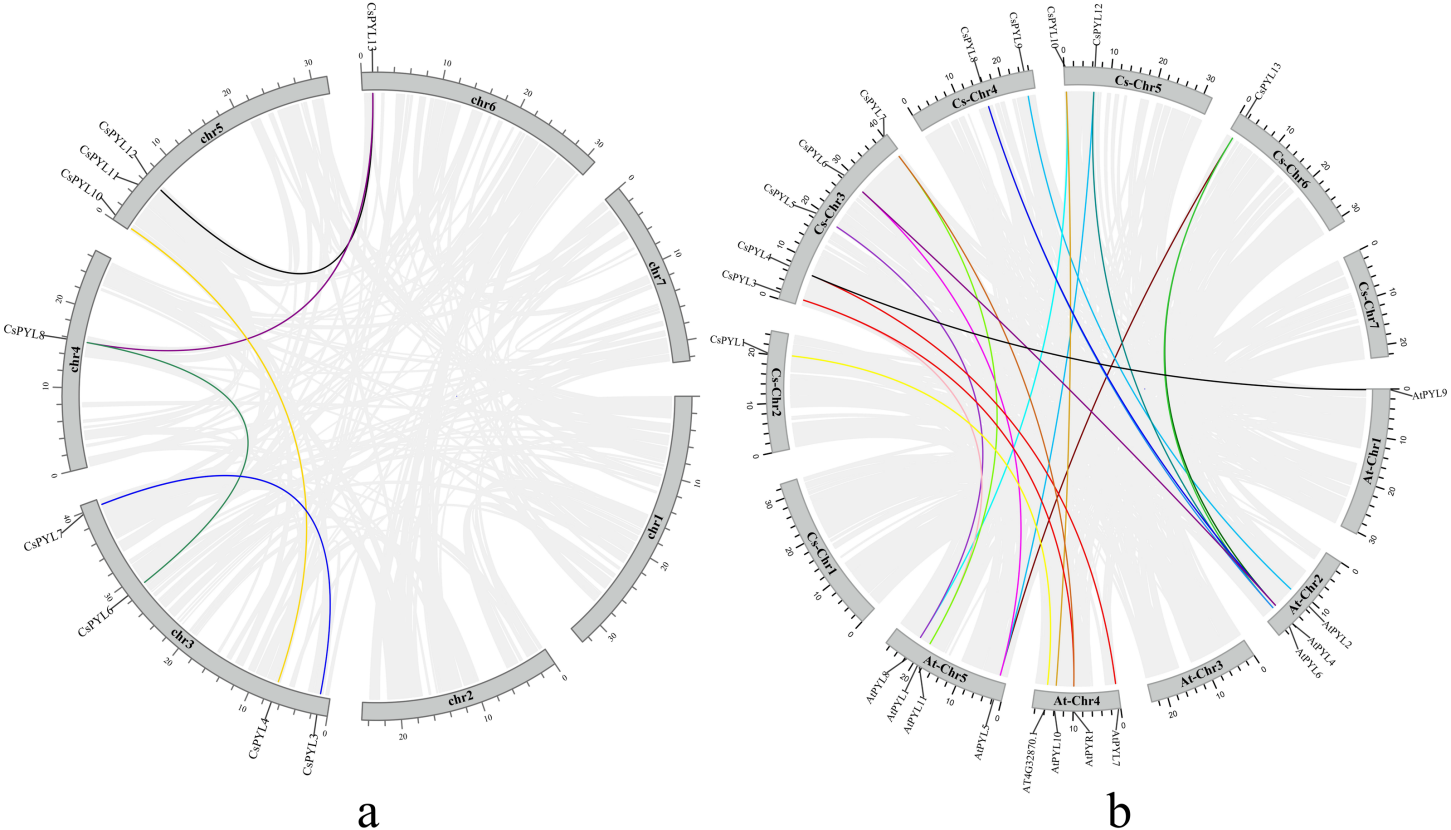
Collinearity analysis of the *PYL* genes family. (A) Collinearity analysis of the *PYL* genes family in cucumber. Chromosomes 1–7 are represented by gray rectangles. The gray lines indicate synteny blocks in the cucumber genome, while the lines of different colors between chromosomes delineate segmental duplicated gene pairs. (B) Collinearity analysis of the *PYL* gene family between cucumber and *Arabidopsis thaliana*. Gray lines denote the collinear blocks between cucumber and Arabidopsis genomes and thelines of different colors denote the syntenic gene pairs of *PYLs*. Gray rectangles represent respectively the cucumber chromosomes (1–7) and *Arabidopsis* chromosomes (1–5).

Using Mcscanx and TBtools to analyze the segmental duplication events, it was found that there were 5 pairs of collinearity genes in cucumber (Fig. 2A), including 4 segmental duplication events between different chromosome, containing (*CsPYL4*/*CsPYL10*, *CsPYL6*/*CsPYL8*, *CsPYL8*/*CsPYL13*, *CsPYL12*/*CsPYL13*), the other 1 duplication events within the same chromosome, including (*CsPYL3*/*CsPYL7*).

**Figure 3:**
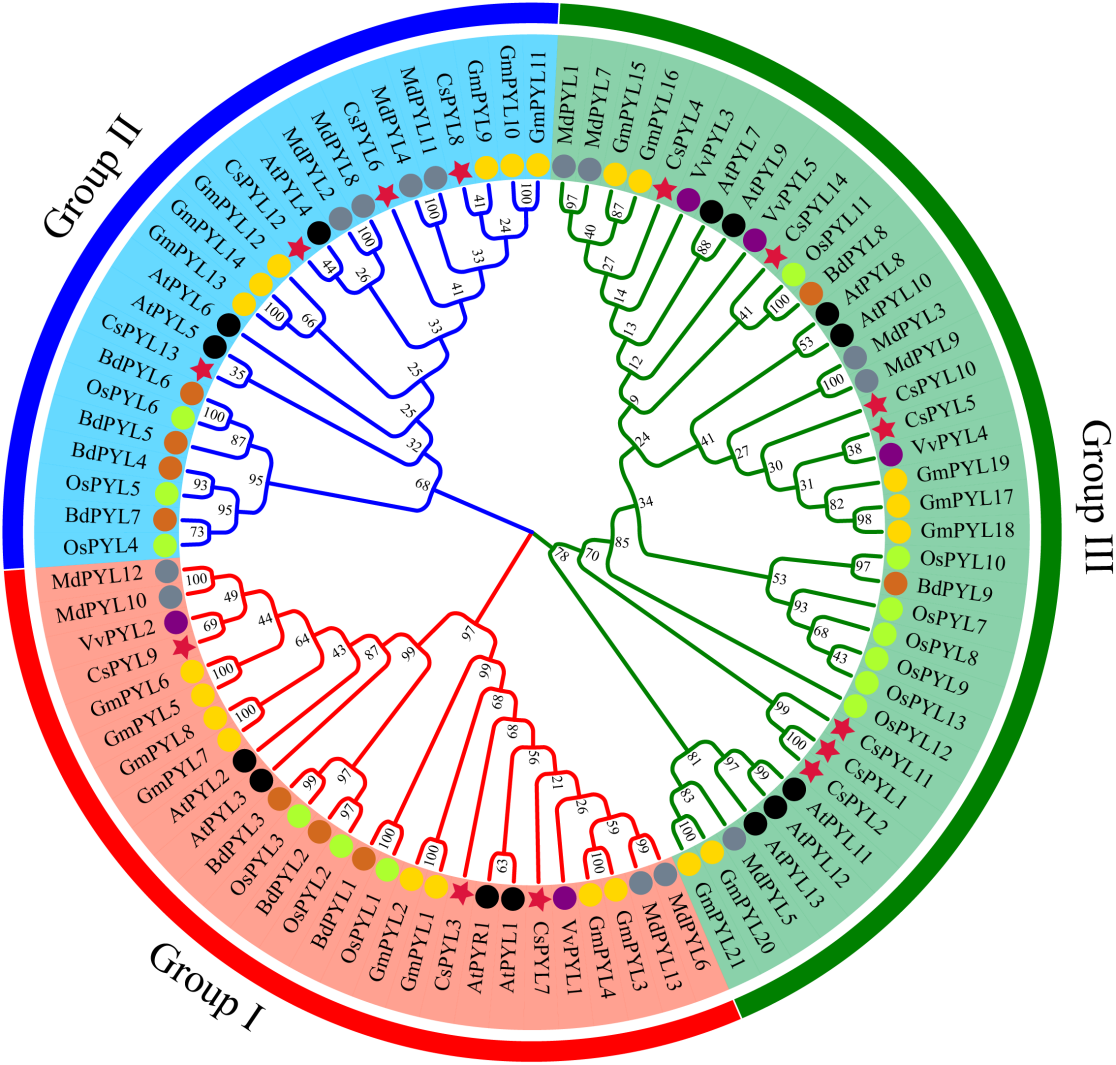
*PYL* phylogenetic trees of seven plants. Red circles represent *C. sativus*, black circles represent *A. thaliana*, blue circles represent *M. domestica*, green circles represent *O. sativa*, yellow circles represent *G. max*, purple circles represent *V. vinifera*, and pink circles represent *B. distachyon*.

**Figure 4:**
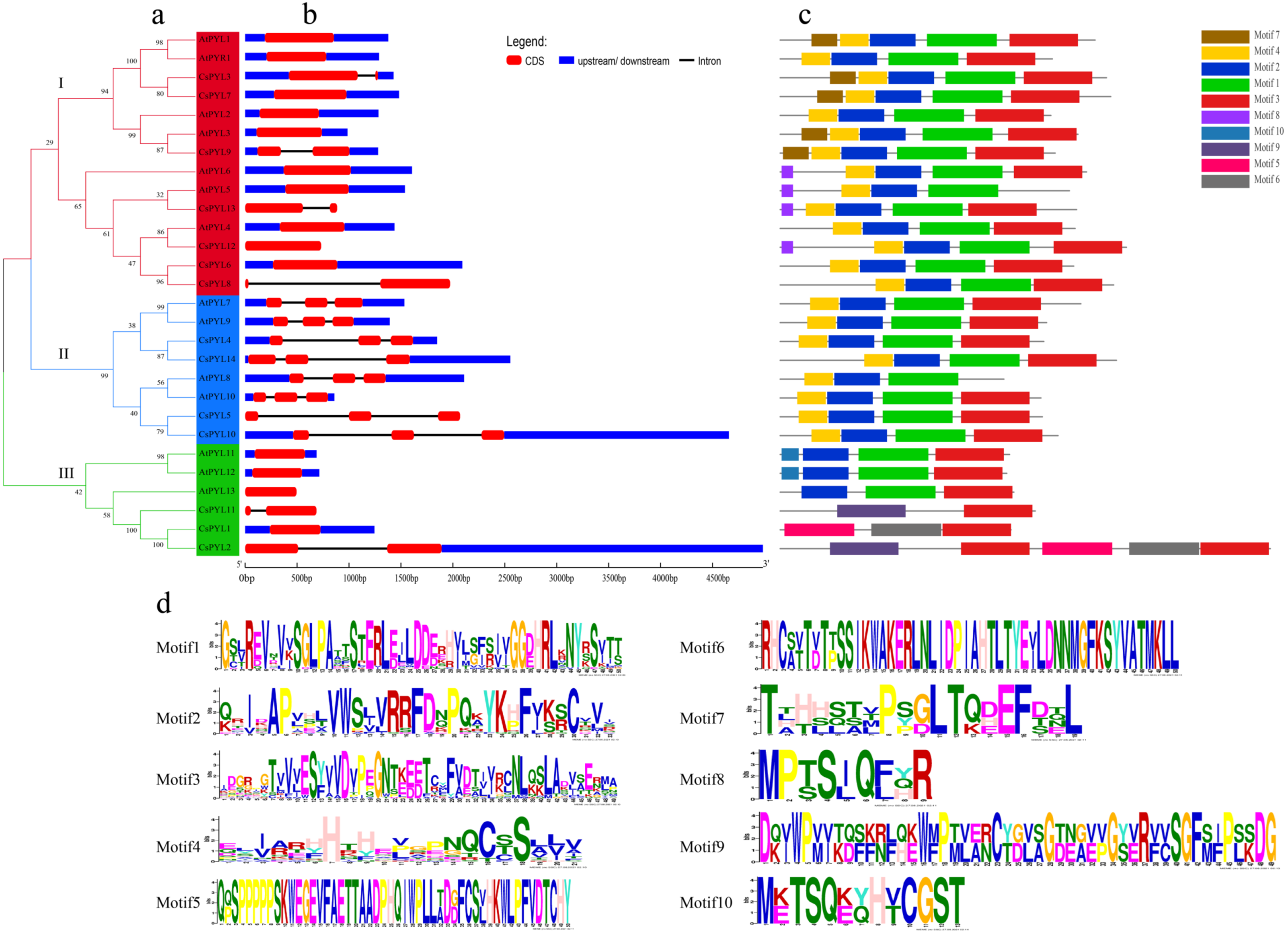
Phylogenetic relationships, gene structure and conserved motifs of *PYL* genes. (A) Phylogenetic relationship of *PYLs* from in *Arabidopsis* and cucumber. Tree was constructed by the Maximum likelihood method. (B) Exon/intron architectures of *PYLs*. Red colour boxes indicate exons and lines represent introns. (C) Distributions of conserved motifs in PYL proteins. Motifs are indicated by 10 different colour boxes. (D) The pattern identification of ten conservative sequences.

Subsequently, we also explored the collinearity relationships between the cucumber *PYL* genes and *Arabidopsis* (Fig. 2B). There were 19 collinear gene pairs between 11 *CsPYLs* and 11 *AtPYLs*. According to the collinearity relationship between *CsPYLs* and *AtPYLs*, most collinear gene pairs were distributed on At-Chr 2, 4, 5 and Cs-Chr 3-5. In addition, the collinear relationship between most *CsPYLs* and *AtPYLs* was one-to-many, such as the collinear relationships between *AtPYL1* and *CsPYL3, 7*; *AtPYL4* and *CsPYL6, 8, 13.* The genes with collinear relationship in our study belong to the same group in the phylogenetic tree (Fig. 3) and were very similar to their gene structure and conserved motifs (Fig. 4), such as *AtPYL1*, *AtPYR1* and *CsPYL3*, *7* which all have upstream/downstream sequence and two exons (except *CsPYL3*).

### Analysis of d_N_/d_s_ values of *PYLs* in cucumber, cucumber and *Arabidopsis*

To further investigate the divergence and selection in duplication of *PYL* genes, the non-synonymous substitution rate (d_N_), synonymous substitution rate (ds) and d_N_/d_s_ values were evaluated for the homologous gene pairs among cucumber, cucumber and *Arabidopsis* (Table 3). When d_N_/d_S_ > 1 is the positive selection, d_N_/d _S_ = 1 is the neutral selection, 0 < d_N_/d_S_ <1 is purifying selection (Yadav et al., 2015). The d_N_/d_s_ value of all cucumber gene pairs was less than 1. Similarly, the d_N_/d_s_ value of all collinear gene pairs in cucumber and *Arabidopsis* was less than 1. These data suggest that these genes were mainly under the purifying selection during evolution and could help to maintain the basic function of this gene.

### Phylogenetic analysis of the *CsPYLs* family

In order to determine the evolutionary relationship of *PYL* genes, a phylogenetic tree was constructed using the 14 *CsPYL* genes from *C. sativus*, 14 *AtPYLs* from *A. thaliana*, 13 *OsPYLs* from *O. sativa*, 21 *GmPYLs* from *G. max*, 5 *VvPYLs* from *V. vinifera,* 9 *BdPYLs* from *B. distachyon* and 13 *MdPYLs* from *M. domestica* (Supplemental Information 4). A total of 89 *PYL* genes of the five plants were divided into three groups (Fig. 3), namely group I-III, which consisted 27, 24 and 38 members respectively. Every group contained *PYLs* from eudicots and monocots, hinting that these *PYLs* generated before the divergence from eudicots and monocots.

**Table 3:** Selective pressure analysis of *PYL* genes family.

**species**	**A pair of genes**	**S**	**N**	**ds**	**d**_**N**_	**d**_**N**_ **/d**_**s**_
**cucumber**	*CsPYL3*-*CsPYL7*	177.3	482.7	3.558	0.2446	0.0688
	*CsPYL6*-*CsPYL8*	136	470	57.1029	0.2383	0.0042
	*CsPYL4*-*CsPYL10*	137.7	417.3	9.8761	0.1667	0.0169
	*CsPYL12*-*CsPYL13*	156.5	389.5	44.7183	0.2544	0.0057
	*CsPYL8*-*CsPYL13*	141.3	413.7	50.1652	0.3002	0.006
**cucumber and** ***Arabidopsis***	*AtPYL1*-*CsPYL3*	170.2	474.8	48.4185	0.3046	0.0063
	*AtPYL1*-*CsPYL7*	168.7	485.3	49.5664	0.2856	0.0058
	*AtPYL2*-*CsPYL9*	127.7	436.3	2.3335	0.2376	0.1018
	*AtPYL4*-*CsPYL6*	138.6	470.4	56.0232	0.3216	0.0057
	*AtPYL4*-*CsPYL8*	136	473	6.4985	0.2768	0.0426
	*AtPYL5*-*CsPYL6*	139.1	430.9	52.2168	0.3448	0.0066
	*AtPYL5*-*CsPYL12*	163.2	442.8	47.2935	0.3561	0.0075
	*AtPYL5*-*CsPYL13*	133.2	400.8	51.1961	0.3025	0.0059
	*AtPYL6*-*CsPYL8*	127.2	472.8	60.1089	0.3307	0.0055
	*AtPYL6*-*CsPYL12*	167.2	474.8	48.8969	0.3593	0.0073
	*AtPYL6*-*CsPYL13*	140.1	408.9	48.2391	0.2968	0.0062
	*AtPYL7*-*CsPYL4*	130.4	424.6	3.572	0.1962	0.0549
	*AtPYL8*-*CsPYL5*	139.2	412.8	2.3447	0.1336	0.057
	*AtPYL8*-*CsPYL10*	122.4	441.6	3.7946	0.1269	0.0334
	*AtPYL9*-*CsPYL4*	126.9	416.1	3.1501	0.1713	0.0544
	*AtPYL10*-*CsPYL10*	129.2	416.8	2.4261	0.1965	0.081
	*AtPYR1*-*CsPYL3*	150.1	416.9	25.5091	0.2497	0.0098
	*AtPYR1*-*CsPYL7*	152.3	420.7	48.2234	0.2499	0.0052

### Gene structure and conserved domains analysis of *CsPYLs*

To understand the structure of the cucumber *PYL* gene family, we analyzed the phylogenetic relationship of the *CsPYL* and *AtPYL* proteins (Fig. 4A), gene structure (Fig. 4B) and conserved motif (Fig. 4C). The 14 *CsPYL* genes were divided into three groups. In the group I, all the *PYL* genes included one or two exons. *CsPYL3*, *9*, *13* and *8* have two exons, and both have one intron with different lengths, among which *CsPYL8* and *13* have no upstream and downstream gene sequences. In the group II, all *PYL* genes have three exons and two introns, only *CsPYL5* has no upstream and downstream sequences. In the group III all *PYL* genes have one or two exons, only *AtPYL13* and *CsPYL11* has no non-coding regions. *CsPYL12* had the longest fragment length that was more than 4,500 bp.

The protein sequences of *CsPYLs* and *AtPYLs* were analyzed by MEME website, and a total of 10 conserved motifs were identified (Fig. 4C). All the *PYL* members contain 2 to 5 conserved motifs. Compared with other genes, *CsPYL* genes in group III had no motif 1 and 2, while *AtPYL8* and *AtPYL5* did not have motif 3. Except all *PYL* genes in the group III other *PYL* genes have motif 4. This indicates that PYL proteins have highly conserved amino acid residues. Besides the common motif, *PYL* genes in each group have its own unique motif, such as *AtPYL1*, *CsPYL7*, *CsPYL3*, *AtPYL2*, *CsPYL9*, *AtPYL3* in group I contain motif 7. *CsPYL1*, *CsPYL2* in group III contained motif 5, motif 6 and motif 9 which were not found in other groups. From these, the proteins classified into the same group share similar motif characteristics, suggesting functional similarities among members, while presence of unique motifs might carry out unique/specialized biological functions.

### Cis-Element analysis of the *CsPYLs* promoter in cucumber

**Figure 5:**
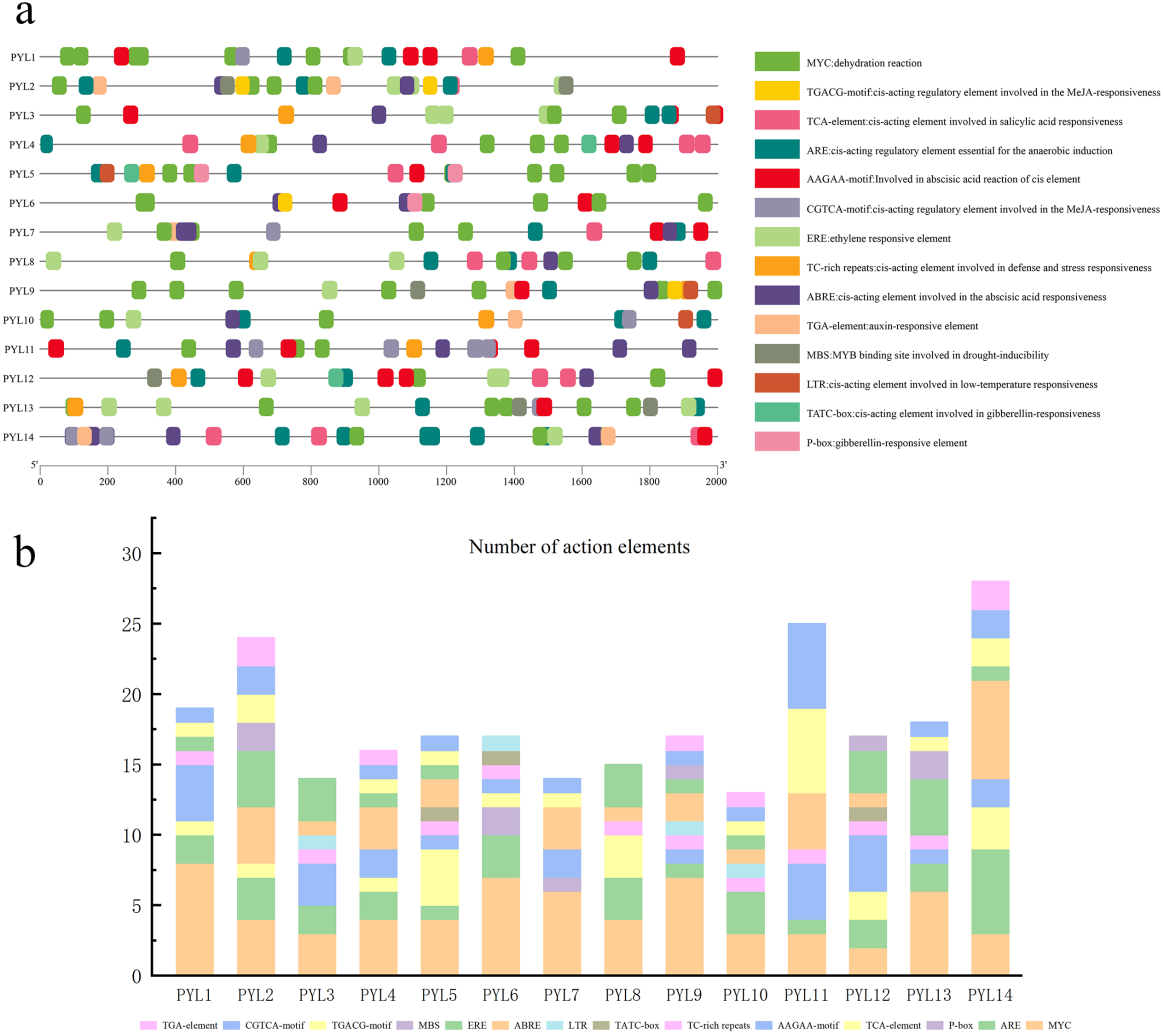
Putative cis-elements existed in the 2 kb upstream region of cucumber *PYL* genes. (A) The elements which respond to hormones are displayed in differently coloured boxes. The homeopathic elements represented by different color boxes and their names and functions. (B) Cucumber *PYL* gene cis-acting elements and number statistics.

The 2,000 bp sequence before the start codon was selected to predict cis-acting elements. As is shown in Fig. 5A, and it was found that the regulatory elements of the *CsPYLs* were very abundant in number and variety, mainly analyzes the hormone regulation and stress-related elements. Among the 14 *CsPYLs* promoters, contain 4 stress-related elements: MYC, TC-rich repeats, MBS, LTR. Additionally, there were 6 hormone-related cis-acting elements predicted, for instance, CGTCA-motif and TGACG-motif are related to MeJA responsive element; TATC-box and P-box are related to Gibberellin-responsiveness responsive element; AAGAA-motif and ABRE are related to abscisic acid response element; TCA-element are related to SA responsive element, as well as ethylene response element (ERE) and auxin response element (TGA-element).

The promoter regions of 14 *CsPYL* genes contain at least 6 acting elements, and all *CsPYL* gene contains MYC and ARE elements (except for *CsPYL7*), among which MYC has the largest number. It can be seen from the statistical table (Fig. 5B) that *CsPYL14* has the largest number of cis-acting elements, followed by *CsPYL2* and *CsPYL11*.

### Tissue-specific expression of *CsPYL* genes in cucumber

**Figure 6:**
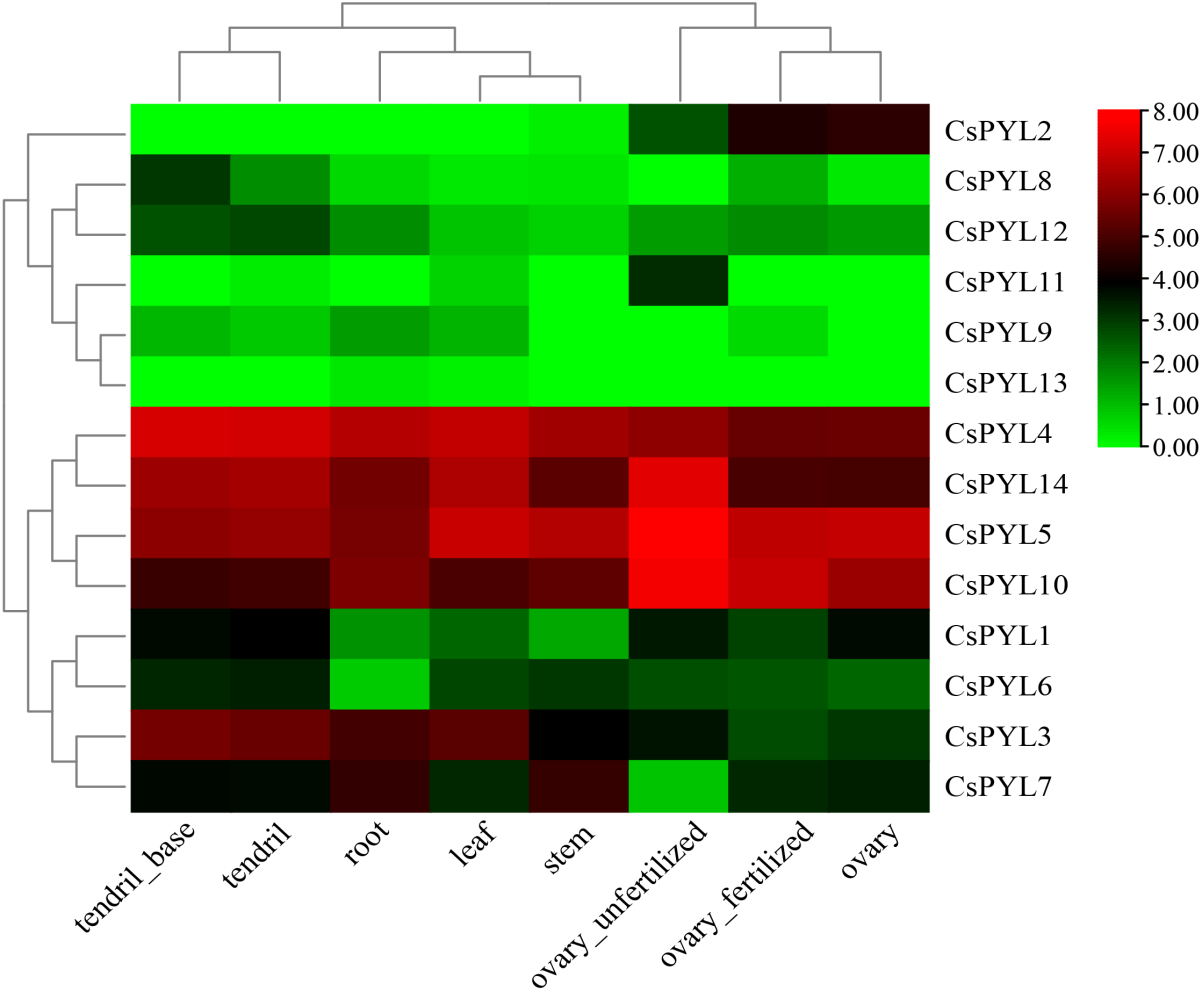
Tissue-specific digital expression profifiles of the *CsPYL* genes in cucumber. The *CsPYL* genes were listed at the right of the expression array, and the expression values mapped to a color gradient from low (green) to high expression (red) are shown at the right of the figure.

In order to better understand the role of *CsPYL* genes in cucumber growth and development, transcriptome data from cucumber database were used to analyze their expression in different tissues. Expression patterns of 14 *CsPYL* genes in different tissues were constructed based on transcriptome data (Fig. 6). The expression of * CsPYL1, CsPYL3-7, CsPYL10* and *CsPYL14* in different eight tissues were high. On the contrary, the expression levels of *CsPYL9, CsPYL11* and *CsPYL13* in tissues were very low, and *CsPYL13* was almost not detected, while *CsPYL11* is only elevated in unfertilized ovaries. The expression of *CsPYL2* was high in the fertilized ovaries, unfertilized ovaries and ovary, but even hardly detected in other parts. The spatial variations in the expression of *CsPYL* genes in different tissues indicate that they may participate in different stages of cucumber growth and development processes.

### Response of *CsPYL* genes expression to various abiotic stresses and ABA treatment

PYLs, as ABA receptors, might play a vital function in abiotic stress signaling pathways. To further understand the expression of *PYL* genes under different abiotic stresses, qRT-PCR was used to analyze the expression of 14 cucumber *CsPYL* genes upon various abiotic stress treatments (including ABA, NaCl, PEG). Raw CT values were placed in (Supplemental Information 5).

**Figure 7:**
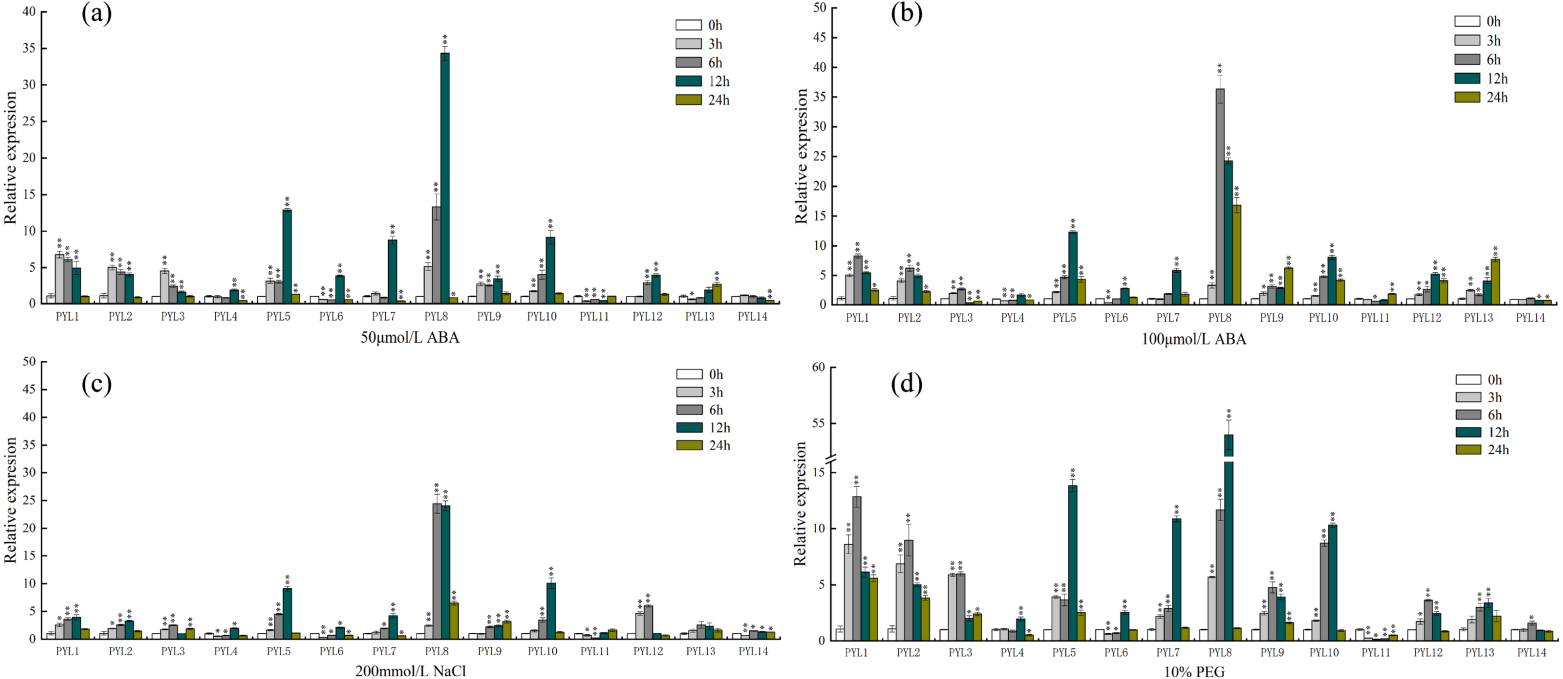
Expression profiles of *CsPYL* genes in response to various stress. (A) A total of 50 µmol/L ABA, (B) 100 µmol/L ABA, (C) 200 mmol/L NaCl, and (D) 10% PEG . The relative expression levels of *CsPYL* genes were analyzed at 3, 6, 12 and 24 h of treatments as compared with their values at 0 h. The gene relative expression was calculated using the 2^−ΔΔCt^ method with CsActin as an internal control, and value represents mean ±SE of three biological replicates. Asterisks indicated values that are significantly different from CK (0 h) (* *p* < 0.05, ** *p* < 0.01, one-way ANOVA).

As shown in Fig. 7A, under the treatment of 50 µmol/L ABA, the expression of *PYL8* was up-regulated at all time points (except 24 h), and reached the highest level at 12 h, which was 30 times higher than that of 0 h (control check). Compared to the 0 h, the relative expression quantity of *PYL1*-*3*, *PYL5*, *PYL9* and *PYL10* were up-regulated about 3 h, 6 h and 12 h. However, after 24 h, the expression level of this genes was close to 0 h. On the Contrary, compared with 0 h, *PYL11* relative expression was down-regulated about 3 h, 6 h and 12 h, then increased slowly, and reached the 0 h level at 24 h. Expression of *PYL14* continually decreased in response to 50 µmol/L ABA. When ABA concentration doubled (Fig. 7B), the expression levels of *PYL1*, *PYL2*, *PYL5*, *PYL8-10*, *PYL12* and *PYL13* were all up-regulated in the four time periods, especially *PYL8* at 24 h, which was 36 times higher than 0 h. *PYL11* showed the same changes as 50 µmol/L ABA treatment, but decreased slightly at 3 h, 6 h and 12 h, and up-regulated at 24 h, which was 1.8 times of 0 h. Only the expression of *PYL14* was slightly down-regulated at all time points.

After 200 mmol/L NaCl treatment for (Fig. 7C) 12 h, the relative expression levels of *PYL5*, *PYL8* and *PYL10* up-regulate and were 9, 24 and 10 times higher than that of 0 h respectively. Compared with 0 h, except *PYL4*, *PYL6* and *PYL11* were down-regulated at 3 h and 6 h, other genes were up-regulated in varying degrees. The relative expression of *PYL14* was basically unchanged. Most of the genes were up-regulated after 10% PEG (Fig. 7D), especially *PYL8* at 12 h, which was 53 times higher than 0 h. It can be seen that the relative expression of *PYL* genes under 10% PEG treatment is higher than other treatments, such as *PYL1*-*5*. This result showed that many *CsPYL* genes (especially *PYL8*) were likely to play critical roles in the abiotic and hormonal stress signaling transduction pathways.

## Discussion

Studies show that ABA can not only regulate the growth and development of plants, but also improve the stress resistance of plants. ABA is perceived by *PYL* family of receptors, which are the largest plant hormone receptor family known (Dupeux et al., 2011), *PYLs* play an important role in ABA signal transduction. In our study, we found 14 *CsPYL* genes, which was almost equal number of *AtPYL* (Park et al., 2009) from *Arabidopsis*. Chromosome mapping showed that (Fig. 1) *PYL* genes distributed on 6 chromosomes, and most of them were distributed on chromosome 3, and there was no clustering phenomenon. However, *PYL* gene of rice and *Brassica napus* were partially clustered (Di et al., 2018; Yadav et al., 2020), which may indicate that there were different trajectories in the evolution of different plants. Segmental duplication, tandem duplication and transposition events are the main reasons for gene family expansion (Kong et al., 2007). Synteny analysis suggested that most *CsPYL* genes were involved in syntenic blocks (Fig. 2A), indicating that segmental duplication events play major roles in the expansion of the *CsPYLs* in cucumber. The collinear relationship (Fig. 2B) between cucumber and *A. thaliana PYL* genes was analyzed, and 20 pairs of collinear genes were found between cucumber and *A. thaliana*. Also, the d_N_/d_S_ values of these gene replication pairs were all less than 1, indicating that they had been purified and selected to predict their high conservatism in the evolution process (Table 3).

According to the phylogenetic tree analysis (Fig. 3), *PYL* genes of *C. sativus*, *A. thaliana*, *O. sativa, G. max, V. vinifera, B. distachyon* and *M. domestica* were divided into three groups, which were consistent with the grouping of rice (Yadav et al., 2020), *Brassica napus* (Di et al., 2018), cotton (Zhang et al., 2017) and rubber trees (Guo et al., 2017), but were different from the groupings of the apple which were divided into four groups (Hou et al., 2020). This difference may be due to the diversity of different species sequences. Moreover, we observed cucumber *PYLs* clustered more closely with dicotyledonous plants *PYLs* than monocotyledonous plants, in agreement with the evolutionary relationships among these plants.

Meanwhile, Fig. 4A showed that the groups of the cluster analysis of *CsPYL* and *AtPYL* also had three. The exon/intron structure of genes is an important marker to reveal the evolutionary relationship between members of the gene family (Long et al., 2003). The gene structure (Fig. 4B) showed that that all *CsPYL* genes have one to three exons, only *CsPYL1, CsPYL6* and *CsPYL7* have one exon, and they all have two non-coding regions and no introns. The members in different groups are similar in structure, some closely related *PYL* genes have similar lengths of exons, such as all members in group II, suggesting that these genes are highly conserved during evolution. The conserved domains of *CsPYL* and *AtPYL* genes analysis showed that motif 3 was found in all *PYL* genes (except *AtPYL8*, *5*). Besides *CsPYL2*, *CsPYL1* and *CsPYL11* in group III, motif 1 and motif 2 were found in all *PYL* genes. This indicates that motif 1, 2 and 3 are highly conserved. HHpred analysis to affirm if the motifs obtained from the MEME analysis are similar to any of the known protein motifs (Table S2), it was found that all three motifs belong to the Polyketide cy-clase/dehydrase family, which are enzymes involved in polyketide synthesis (Iyer, Koonin & Aravind, 2001). It was observed that these novel motifs did not show any significant similarity with the known motifs, which was consistent with the findings in rice (Yadav et al., 2020). This also indicates that all the identified *CsPYLs* have typical subfamily features and the proteins classified into the same subgroup share similar protein motifs.

Understanding the protein’s subcellular location information (Table 2) may provide us with the necessary help to infer the biological function of the protein, *CsPYLs* would mainly locate in nucleus, cytoplasm and chloroplast, so it was speculated to be related to photosynthesis, respiratory action and cell growth and development. The plant tissue expression specificity (Fig. 6) showed that *PYL* genes had varying degrees tissue-specific expression. In rubber trees, *Brassica napus* and rice *PYL* genes also show preferential expression in different tissue types. (Guo et al., 2017)(Di et al., 2018)(Yadav et al., 2020). Our study showed that only *CsPYL13* and *CsPYL9* were low or not expressed in all tissues. The expression levels of *CsPYL2* were higher in fertilized ovary, unfertilized ovary and ovary, *CsPYL8* and *CsPYL12* were higher in the tendril base, and *CsPYL11* were higher in unfertilized ovary, but the expression levels of these genes were lower in other organs. Other genes, such as *CsPYL4, 5, 10,* and *14* were highly expressed in all tissues. In a previous study by Wang et al. (2012b), their research found that, the Cs*PYL2* may involve in transducing ABA signal in fruit and regulating fruit development and ripening. Under drought stress condition in cucumber seedlings. *CsPYL1* and *CsPYL2*, were sensitive and up-regulated in root, stem and leaf; Cs*PYL3* showed a low sensitivity and were down-regulated in root and stem. In our study, *CsPYL10*, *CsPYL13* and *CsPYL14* correspond to *PYL1*, *PYL3* and *PYL2* in the study of Wang et al., and *CsPYL10, CsPYL14* were highly expressed in different tissues of cucumber. On the contrary, *CsPYL13* was almost not expressed in different tissues, which was consistent with their study.

When plants are under various stresses, these stresses signals will activate transcription factors, which combine cis-acting elements to stimulate the expression of related genes. (Mishra et al., 2014; Xing et al., 2018). In plant development and resistance to various stresses, cis-acting elements are important regulators of hormone response (Nishimura et al., 2010). In this study, among all the functional elements, MYC element exists in every *CsPYL* gene, and drought responsive elements are also abundant in *PYL* genes of rubber trees and cotton (Zhang et al., 2017; Guo et al., 2017). The results of qRT-PCR (Fig. 7D) suggested that *CsPYLs* expression were higher than 0 h with 10% PEG treatment (Except *PYL4*, *11*, *14*), indicating that most of *CsPYLs* may had drought resistance, which was similar to the results of cis-acting element. In addition, the *CsPYLs* also contained other hormone related cis-acting elements, such as a large number of ABA response sites (AAGAA-motif, ABRE).

In *Arabidopsis*, over-expression of *PYL4* and *PYL9* can enhance the drought resistance (Pizzio et al., 2013; Zhao et al., 2016)*[18,19].* From the phylogenetic tree (Fig. 3), *AtPYL4* and *CsPYL12* are in the same group, *AtPYL9* and *CsPYL14*, *CsPYL4* in the same group. After different treatments (Fig. 7), *CsPYL14* and *CsPYL4* were almost not expressed, while *CsPYL12* was up-regulated, but it was not obvious compared with other genes. This suggests that the relative expression levels of *PYL* gene may vary among different species. Our study shows that, under 50 µmol/LABA treatment (Fig. 7A), the expression levels of *CsPYL5, 7, 8* and *10* were up-regulated for 3 h, 6 h and 12 h relative to 0 h, and *CsPYL8* up-regulation is most obvious. When the concentration of ABA increased (Fig. 7B), the relative expression of some genes were up-regulated at 24 h, such as *PYL1*, *PYL2*, *PYL5*, *PYL8*-*10,* especially *PYL8*, which was more 10 times than 0 h. It was speculated that when ABA concentration increased, the expression level of *PYL* genes was affected by ABA more lasting. Overall, except *PYL4, PYL11* and *PYL14*, all *CsPYL* genes increased more or less under ABA treatment. This was similar to the result of apple *PYL* genes under ABA treatment (Hou et al., 2020), the expression of all *MdPYLs* except for *MdPYL11* exhibited significant increases by ABA treatment. However, some *GhPYL* genes were down-regulated under ABA treatment in cotton. After treatment with ABA, *GhPYR1-1A*, *GhPYR1-3D*, *GhPYL9-5A* significantly reduced, and *GhPYL9-2D* was not influenced by ABA treatment (Zhang et al., 2017; Chengzhen et al., 2017). This also indicates that *PYL* genes in different crops have different responses to ABA during the evolutionary process and a great number of *CsPYLs* have different responses to ABA. Under salt stress (Fig. 7C) and drought stress (Fig .7 d) *CsPYL5, 8, 10* were obviously up-regulated and the relative expression of *PYL* genes with 10% PEG treatment is higher than other treatments, such as *PYL1*-*5* and *PYL8*. But in rice, except for *OsPYL4,* all *PYLs* were down-regulated under drought stress and *OsPYL12* was unaltered. In *Arabidopsis*, overexpression of *AtPYL4*, *5*, *7*, *8* and *13* respectively enhanced drought tolerance (Lee et al., 2015; Yadav et al., 2020; Zhao et al., 2016). However, in the study of duodecuple mutants *PYR1* and *PYL1*-*12* by Zhao et al. (2018), it was found that the duodecuple mutant was extremely insensitive to ABA effects on seedling growth, stomatal closure, leaf senescence, and gene expression. Therefore, *PYL* genes may have a potential effect on improving abiotic stress tolerance in plants. Together, all these results show that many *CsPYLs* were likely respond to ABA, drought, salt treatments, and *CsPYL* genes respond more significantly to drought stress than other treatments.

## Conclusion

We identified 14 *PYL* genes in cucumber, which were distributed on 6 chromosomes, and more members on chromosome 3 (*PYL3*-*7*) and 5 (*PYL10*-*12*). Motif 3 was found in all *CsPYL* genes, and its structure was conserved. The analysis of cis-acting elements showed that there were many elements in cucumber *PYL* gene responding to abiotic stress and hormones, and drought responsive element MYC was present in all *CsPYL* genes. Furthermore, qRT-PCR analysis showed that *CsPYLs* (especially *PYL8*) may have certain function in resisting abiotic and hormone stresses. This study provided relevant information for the study of *PYL* gene function in cucumber.

## Supplemental Information

10.7717/peerj.12786/supp-1 Supplemental Information 1 The sequences of primers used for qRT-PCR

10.7717/peerj.12786/supp-2 Supplemental Information 2 Similarity of motifs identified by MEME analysis in 10 Cs*PYL* s with the known protein domains as analyzed by HHPred analysis

10.7717/peerj.12786/supp-3 Supplemental Information 3 The amino acid substitution model

10.7717/peerj.12786/supp-4 Supplemental Information 4 Seven species of protein sequences

10.7717/peerj.12786/supp-5 Supplemental Information 5 The original CT value of qRT-PCR and supplementary figure of actin CT value

## Additional Information and Declarations

### Data Availability

The following information was supplied regarding data availability: The raw measurements are available in the Supplementary File.
