# Supplementary material for: Genome-wide identification and expression analysis of the cucumber PYL gene family
Source: PeerJ. 2022 Jan 11;10:e12786. doi: 10.7717/peerj.12786 (PMC8759363; doi:10.7717/peerj.12786)
Supplement: Supplemental Information 1 [file peerj-10-12786-s001.docx]

| **Gene Name** | **Forward Primer Sequence (5'-3')** | **Reverse Primer Sequence(5'-3')** |
| --- | --- | --- |
| PYL1 | CCATCGCACATACTCTCACCTACG | CCACGAACGACCAGACGAACTTG |
| PYL2 | CATCGCACACACTCTCACCTACG | CCACGAACGACCAGACGAACTTG |
| PYL3 | CCTCCTTACTTGCTCAGCGTGTTC | CCTTCACTCACCGTACAGCTCTTG |
| PYL4 | TGATGGAAGACCTGGGACGC | CTCTCCGATACGTCCGCCAA |
| PYL5 | AGGCATGACCCTGCTGACAA | CCCTGCACTACACACCTGCT |
| PYL6 | AATGGTCCAAGGTGCCGGAA | ATTTCTTGGACCACGGCGGA |
| PYL7 | GCGACTAGTACAGAGCGGCT | CCAGATCTGACCGTCACGCT |
| PYL8 | ACGTTGGAAGCCTCCGTGAA | GTCGAGAATCTCCAGCCGCT |
| PYL9 | CTTCCGGCATCAACGAGCAC | TCTCCTCCCACCACCCTGAA |
| PYL10 | GCACTGGGAATGGGAATGGG | TTCTCAGCAGGCTCGTTCCT |
| PYL11 | AGAGGCACTGGCATGAGCAT | TGAGTGACCACGGGCCATAC |
| PYL12 | TCTGGACTTCCGGCTGCTTC | TAGGCGATGGTCCCCACCTA |
| PYL13 | GTTCGCCGATTCGACAACCC | TTTACTCGACACCGCTGGCA |
| PYL14 | CCTGGGACACTGGTGGTTGA | GCATCACGGCCATCCTCTCA |
| actin | GCCCTCCCTCATGCCATTCT | TCGGCAGTGGTGGTGAACAT |
