# Supplementary material for: Genome-wide identification and expression analysis of the cucumber PYL gene family
Source: PeerJ. 2022 Jan 11;10:e12786. doi: 10.7717/peerj.12786 (PMC8759363; doi:10.7717/peerj.12786)
Supplement: Supplemental Information 2 [file peerj-10-12786-s002.docx]

**Additional File 5: Table S2**

Similarity of motifs identified by MEME analysis in 10 Cs*PYL*s with the known protein domains as analyzed by HHPred analysis.

| **Motif** | **Hit (PDB id)** | **Prob.** | **E-value** | **SS** | **Target Length** |
| --- | --- | --- | --- | --- | --- |
| **Motif 1** | SlPYL1; ABA, RECEPTOR, SIGNALING, STRESS, Signaling protein; 1.65A {Solanum lycopersicum} | 97.62 | 0.00043 | 6.1 | 232 |
|  | Abscisic acid receptor PYL5; abscisic acid receptor, [Arabidopsis thaliana] | 97.52 | 0.00074 | 6.3 | 223 |
|  | Polyketide cyclase/dehydrase and lipid transport superfamily protein [Arabidopsis thaliana] | 96.66 | 0.0044 | 3.5 | 122 |
| **Motif 2** | SlPYL1; ABA, RECEPTOR, SIGNALING, STRESS, Signaling protein; 1.65A {Solanum lycopersicum} | 98.58 | 1.9e-7 | 3.9 | 232 |
|  | Abscisic acid receptor PYL10; PYL10, ABA-independent PP2C inhibitor, PP2Cs, abscisic acid, ABA receptor, HYDROLASE INHIB | 98.57 | 2.6e-7 | 4.1 | 183 |
|  | Polyketide cyclase/dehydrase; Helix-grip fold, polyketide C9-C14 aromatase/cyclase, linear poly-beta-ketone intermediate, BIOSYNTH | 98.48 | 2.8e-7 | 2.9 | 159 |
| **Motif 3** | Abscisic acid receptor PYL5; abscisic acid receptor, PP2C, HORMONE RECEPTOR; HET: GOL; 2.65A {Arabidopsis thaliana} | 97.2 | 0.0029 | 6.2 | 223 |
|  | SlPYL1; ABA, RECEPTOR, SIGNALING, STRESS, Signaling protein; 1.65A {Solanum lycopersicum} | 96.56 | 0.02 | 6.1 | 232 |
|  | Polyketide cyclase/dehydrase and lipid transport superfamily protein [Arabidopsis thaliana] | 76.72 | 13 | 6.1 | 189 |
| **Motif 4** | TRYPSIN INHIBITOR 3; HYDROLASE-INHIBITOR COMPLEX, MINIPROTEIN SCAFFOLD, KNOTTINS, SERINE PROTEASE INHIBITOR; HET: MES, I | 58.09 | 5.1 | 0.1 | 37 |
|  | CALMODULIN-DOMAIN PROTEIN KINASE 1, PUTATIVE; NUCLEOTIDE-BINDING, SERINE/THREONINE-PROTEIN KINASE, KINASE, TRANSFERASE | 36.6 | 35 | 1.1 | 287 |
|  | Genome polyprotein; HRV, 3C, protease, rupintrivir, HYDROLASE; HET: AG7; 2.05A {Rhinovirus C} SCOP: b.47.1.0 | 35.91 | 33 | 0.9 | 183 |
| **Motif 5** | Abscisic acid receptor PYL10; PYL10, ABA-independent PP2C inhibitor, PP2Cs, abscisic acid, ABA receptor, HYDROLASE INHIB | 98.28 | 0.0000047 | 5.5 | 183 |
|  | ABA receptor RCAR3; abscisic acid, ABA, receptor, phosphatase, stress, complex, PLANT PROTEIN; HET: A8S; 2.1A [Oryza sat] | 98.25 | 0.0000055 | 5.2 | 175 |
|  | Abscisic acid receptor PYL9; Abscisic acid receptor, Drought tolerance, protein phosphatase inhibitor, START/Bet v1 fami | 98.24 | 0.0000061 | 5.3 | 189 |
| **Motif 6** | Bet v I allergen family; Structural genomics, Arabidopsis Thaliana, Center for Eukaryotic Structural Genomics, Protein S | 97.74 | 0.00017 | 4.9 | 122 |
|  | SlPYL1; ABA, RECEPTOR, SIGNALING, STRESS, Signaling protein; 1.65A {Solanum lycopersicum} | 95.07 | 0.21 | 5.7 | 232 |
|  | Abscisic acid receptor PYL5; abscisic acid receptor, PP2C, HORMONE RECEPTOR; HET: GOL; 2.65A {Arabidopsis thaliana} | 93.77 | 0.69 | 5.9 | 223 |
| **Motif 7** | SlPYL1; ABA, RECEPTOR, SIGNALING, STRESS, Signaling protein; 1.65A {Solanum lycopersicum} | 95.24 | 0.024 | 2 | 232 |
|  | UPF3X; NONSENSE MEDIATED MRNA DECAY PROTEIN, RNA-BINDING PROTEIN, NMD, RNP DOMAIN, MIF4G DOMAIN, RNA BINDING PROTEIN; 1. | 93.2 | 0.088 | 1.4 | 91 |
|  | Phosphoprotein; negative strand RNA virus, polymerase, replication, cofactor, viral protein; 2.3A {Vesicular stomatitis | 79.18 | 1.2 | 0.6 | 75 |
| **Motif 8** | Abscisic acid receptor PYL5; abscisic acid receptor, PP2C, HORMONE RECEPTOR; HET: GOL; 2.65A {Arabidopsis thaliana} | 68.97 | 3.3 | 0.6 | 223 |
|  | MANNOSE-6-PHOSPHATE ISOMERASE; ISOMERASE, APO-STRUCTURE, METAL-BINDING; HET: EDO; 1.67A {SALMONELLA TYPHIMURIUM} | 44.08 | 13 | 0.4 | 394 |
|  | Putative presegetalin F1; segetalin biosynthesis, prolyl oligopeptidase, macrocyclase, peptidase, beta-propeller, closed | 41.55 | 18 | 0.5 | 38 |
| **Motif 9** | Lachrymatory-factor synthase; lachrymatory factor, onion, sulfenic acid, ISOMERASE; 1.4A {Allium cepa} | 98.18 | 0.000007 | 4.4 | 157 |
|  | Hydroxynitrile lyase; hydroxynitrile lyase, fern, (R)-mandelonitrile, benzaldehyde, lyase; HET: HBX, MXN; 1.5A {Davallia | 97.98 | 0.000019 | 3.9 | 209 |
|  | SlPYL1; ABA, RECEPTOR, SIGNALING, STRESS, Signaling protein; 1.65A {Solanum lycopersicum} | 97.98 | 0.000018 | 3.8 | 232 |
| **Motif 10** | Glycinamide ribonucleotide transformylase 1; Structural Genomics, RIKEN Structural Genomics/Proteomics Initiative, RSGI, | 59.46 | 7.8 | 0.9 | 229 |
|  | Myb-related protein B; Myb, B-Myb, LIN52, LIN9, MMB, MuvB, CELL CYCLE, CELL CYCLE-DNA Binding complex; HET: SO4, MSE; 2. | 48.24 | 14 | 0.7 | 32 |
|  | Insulin; Human insulin, hormone, glucose metabolism, secretion; 0.92A {Homo sapiens} | 43 | 12 | 0.2 | 30 |
